# Sleep deprivation: a risk for epileptic seizures

**DOI:** 10.5935/1984-0063.20220046

**Published:** 2022

**Authors:** Jason Tyler Dell’Aquila, Varun Soti

**Affiliations:** 1 Lake Erie College of Osteopathic Medicine, Osteopathic Medical Student, 2nd year year - Elmira - New York - United States.; 2 Lake Erie College of Osteopathic Medicine, Assistant Professor of Pharmacology - Elmira - New York - United States.

**Keywords:** Epilepsy, Sleep, Physiology, Gamma-Aminobutyric Acid

## Abstract

There is a well-documented correlation between epilepsy and sleep deprivation. For decades, preclinical and clinical studies have shown that sleep deprivation can lead to an increased risk of epileptic seizures. Additionally, sleep deprivation has been used clinically as a diagnostic tool for epilepsy by triggering epileptiform activity. However, an underlying mechanism for this relationship is yet to be confirmed. Interestingly, a decrease in gamma-aminobutyric acid (GABA)-mediated tonic inhibition has been shown in both epilepsy and sleep deprivation. This review focuses on the role of sleep deprivation in the induction of epileptic seizures and the possible role of reduced GABA receptor expression in the sleep-deprived state.

## INTRODUCTION

Approximately 1% of the world’s population suffers from epilepsy^1^. It is a well-known disease even outside the medical field and research realm^2^. Although there is no known cure, many patients control the frequency of their seizures by taking antiepileptic medications^3^. The disease severity can be variable; some patients only occasionally experience a seizure while others have multiple episodes a week. Although it is commonly associated with brief periods of violent convulsions wherein the person loses consciousness, other types of epilepsy manifest differently^4^. Nonetheless, all epileptic seizures share the common characteristic of arising from an overexcitation of neurons in the brain^3,5^. The pathophysiologic mechanisms leading to a seizure episode consist of synchronous neuronal firing and loss of gamma-aminobutyric acid (GABA)-mediated inhibition^3^.

Epileptic seizures have continually been linked to sleep patterns^6^. Specifically, sleep deprivation has been known as a seizure risk in epilepsy patients for many decades^7-9^. Neurologists have even utilized this relationship clinically to aid the early diagnosis of epilepsy syndromes^10-12^. Aside from its role in inducing seizures, sleep deprivation has many other deleterious effects on people, including impaired cognitive functioning^13-15^. GABA-mediated inhibition is involved in promoting sleep. Indeed, the reduced expression of specific GABA receptors has recently been linked to the sleep-deprived state^16-18^.

Despite recent research studies showing that sleep deprivation can induce seizures, its pathophysiologic mechanisms have not yet been elucidated. This review focuses on the current literature on sleep deprivation as a trigger for epileptic seizures. Additionally, it highlights the need to investigate the possibility that reduced GABA-mediated inhibition could be a mechanism through which sleep deprivation leads to seizures in epileptic patients.

## MATERIAL AND METHODS

**Figure 1:**
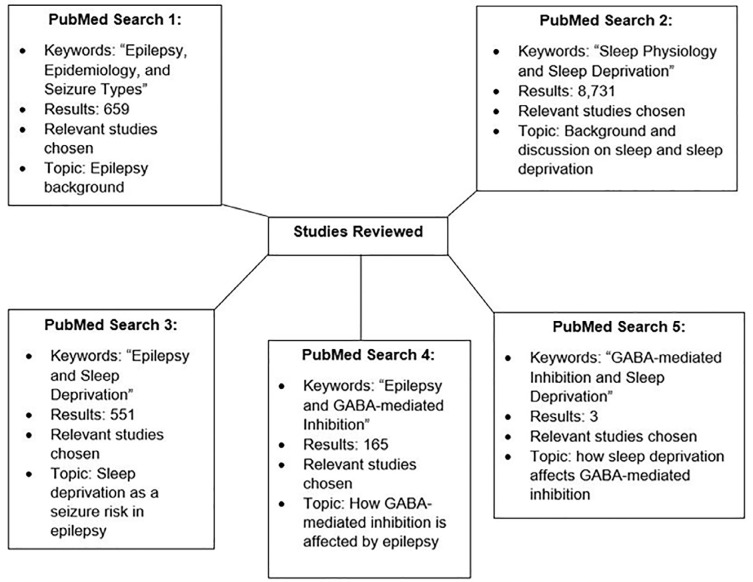
Flowchart illustrating the methodology. Keywords within the same figure box, for example, epilepsy and sleep deprivation in “PubMed Search 3”, were entered into PubMed together to increase the specificity and relevance of the results.

PubMed was utilized for the literature search. This search was conducted between March 2021 - June 2021. Only articles written in English language were selected. Relevant preclinical and clinical studies were included. The keywords “epilepsy, epidemiology, and seizure types,” when entered in the database, yielded 659 results, of which the most relevant studies were selected. The keywords “sleep physiology and sleep deprivation” and “epilepsy and sleep deprivation” resulted in the retrieval of 8,731 and 551 studies, respectively. Out of these, studies that lay within the scope of this review were selected. Additionally, “epilepsy and GABA-mediated inhibition” and “GABA-mediated inhibition and sleep deprivation” led to 165 and 3 results, respectively, from which pertinent studies were chosen. The methodology is illustrated in Figure 1.

## DISCUSSION

### Epilepsy

People are most familiar with tonic-clonic seizures, in which the patient loses consciousness and convulses. However, there are multiple types of epilepsy characterized by different types of seizures^5,19^. In a broad sense, epilepsy can be divided into the generalized type, in which a seizure attack affects both hemispheres of the brain, and the focal type, in which seizures arise from a specific area. Some focal seizures can also generalize. Since they affect only a particular brain region, focal regions manifest with symptoms indicative of the region. For example, temporal lobe seizures can lead to memory loss^20^. Examples of generalized seizures include absence seizures, which typically affect children, and tonic-clonic seizures^5,19,21,22^. Since all episodes of epilepsy stem from the overexcitation of neurons, this review will collectively refer to epilepsy syndromes in the context of sleep deprivation^3,5^. An area of potential future research is the investigation of how sleep deprivation affects the seizure threshold in different types of epilepsy.

Studies have shown that epilepsy has adverse effects on mental well-being and quality of life^23-25^. Depending on the severity of their epilepsy, patients can feel stigmatized by their condition, and numerous people develop depression^23,24^. Epilepsy can lead to more sedentary lifestyles because patients fear having seizures in public. This also would adversely affect health outcomes^25^.

Not all seizures are caused by epilepsy^26^. For instance, patients with post-traumatic stress disorder can sometimes experience psychogenic seizure disorders, termed psychogenic nonepileptic paroxysms (PNES)^27,28^. Such patients are often misdiagnosed as having epilepsy, and research is ongoing on how to effectively differentiate between epileptic and psychogenic seizures^29^. However, PNES are not within the scope of this review, which is entirely focused on examining the relationship between epileptic seizures and sleep deprivation.

### Sleep physiology and sleep deprivation

Despite sleep being a fundamental physiologic function, its mechanisms are complex^16,17,30-33^. Therefore, sleep remains an active area of research. Studies have shown that multiple areas of the brain are involved in promoting sleep^16,30-33^. The hypothalamus is a central regulator of the sleep cycle; specifically, the suprachiasmatic nucleus has been revealed to generate circadian rhythms that determine sleep cycles^30,31^. The brainstem contains a reticular activating system of neurons that is vital in promoting the wakeful state^16,32^. This system must therefore be inhibited for the sleep state to manifest. Additionally, the thalamus, which acts primarily as a relay center for sensory signals traveling from the periphery, is very active during the awake state and rapid-eye-movement (REM) sleep stages^33^. Finally, GABA, the primary inhibitory neurotransmitter in the central nervous system, has been shown to promote sleep^16,17,34,35^.

The sleep cycle can be broadly divided into REM sleep and non-rapid-eye-movement (NREM) sleep^16-18,36,37^. REM sleep is characterized by less synchronization of neuronal firing and more significant brain activity than NREM sleep^17^. NREM sleep can be further divided into the N1 - 3 stages, in which the waveforms become progressively more synchronized and of higher amplitude on electroencephalogram (EEG)^18^. On the other hand, the waveforms of REM sleep appear similar to those of the awake state such as when the person is actively concentrating^17^. There is also a difference in the types of waves characterizing these two stages of sleep. While REM sleep consists of high frequency, low voltage beta waves, the late- -stage of NREM sleep is characterized by very high amplitude, synchronized delta waves^17,18^. Due to the higher levels of synchronization in NREM sleep, this stage has been proven to promote epileptic seizures, while REM sleep is a seizure suppressor^38^.

A plethora of studies have shown that sleep deprivation has specific detrimental health effects. Cognitive functioning is significantly impaired in sleep-deprived patients^13-15^. A meta- analysis revealed that sleep-deprived human subjects displayed decreased mood affect, mental, and motor performance relative to controls^13^. Sleep deprivation also changes EEG waveforms^39,40^. One study found substantial delta wave activity localized in the frontal and parietal lobes in sleep-deprived patients^40^. Since delta waves are synchronous and constitute a seizure risk in epilepsy, these findings implicate a potential mechanism for sleep deprivation lowering the seizure threshold. Future studies should further explore this and determine whether if this pattern can also be found in other brain regions, such as the temporal lobe. As 35% of adults fail to achieve the recommended seven hours of sleep per night^18^, further research into the consequences of sleep deprivation has relevance to patients with and without epilepsy.

### Sleep deprivation as a seizure risk in epilepsy

For many years, it has been apparent that sleep deprivation has a relationship to epileptic seizures^6^. In a questionnaire-based study, people with epilepsy commonly reported sleep deprivation as one of the antecedents of their seizures^41^. Conducted in 1998, this research provided a subjective correlation between sleep deprivation and an increased risk of epileptic seizures. Additionally, there is also a wealth of objective evidence for this relationship. Using transcranial magnetic stimulation one study found that sleep deprivation increases cortical excitability in patients with epilepsy^42^. Several other studies have found EEG evidence of increased epileptiform discharges (EDs) in sleepdeprived subjects with epilepsy^8,9^. In a research study on juvenile myoclonic epilepsy (JME), an idiopathic type of epilepsy that affects children, it was discovered that sleep deprivation increased EDs and paroxysms in such patients^43^.

One potential confounder of these study findings is that sleep deprivation could cause the patients to fall asleep during the study, and the increased EDs were related to being in the sleeping state. Previous studies have sought to address this by controlling for the sleeping state^7,44^, demonstrating that sleep deprivation-induced sleep incited more EDs than natural sleep measured on routine EEG^44^. Similarly, more research needs to be done explaining whether sleep deprivation alone induces more EDs than drug-induced sleep in epileptic patients.

Sleep deprivation has been used clinically for years to produce EDs and aid in the diagnosis of epilepsy. Indeed, a 2010 study showed sleep deprivation helps uncover epileptiform activity, aiding in the diagnosis of epilepsy in children^10^. Other studies have demonstrated the application of sleep deprivation in the diagnosis of epilepsy^11,12^. However, there are discrepancies among the findings of some of these studies. For example, a recent study showcased that sleep deprivation EEG (SD-EEG) was a sensitive diagnostic tool for idiopathic generalized but not focal epilepsy^45^. Conversely, a subsequent empirical evaluation revealed that SD-EEG is an efficacious tool for diagnosing epilepsy^46^. These disparities have been attributed to a wide variance in study methodologies^47^. Future research studies must control for such factors.

### Loss of GABA-mediated tonic inhibition as a potential link between sleep deprivation and epilepsy

GABA is critical in maintaining the integrity of the central nervous system through its inhibitory effects^48^. It functions through two different sets of receptors - GABAA and GABAB^34^. When activated, GABAA receptors allow chloride ions to enter the cell, leading to decreased membrane potential and, consequently, neuronal inhibition. These receptors are *heteropentameric,* consisting of five subunits. Although these subunits are variable, a typical GABAA receptor consists of two alpha (a) subunits, two beta (p) subunits, and a variable fifth subunit, which could be a gamma, delta^6^, epsilon, theta, or pie^49,50^.

GABA receptors mediate two different types of inhibition - phasic and tonic^51^. Phasic inhibition is fast-acting and transient in nature. Once the inhibition is over, GABA is removed from the synaptic cleft by uptake into glial cells and presynaptic neurons, where it is catabolized by GABA transaminase^34^. By contrast, tonic inhibition is much longer in duration and is mediated by receptors outside the synapse^50^. This type of inhibition counterbalances the overexcitation of neurons, implicating a role in seizure prevention^34^. Indeed, drugs that facilitate the action of GABA, known as GABA agonists, can help to suppress seizures, while GABA antagonists can precipitate them^52-54^.

Studies examining human epileptic brain tissue have generally found decreased expression of GABAA receptors^55,56^. Interestingly, specific subtypes of GABAA receptors, 6-GABAA and a5-GABAA, have garnered the attention of researchers as pertains to epilepsy and sleep deprivation^18,57^. These receptors are essential for generating tonic inhibition in many brain regions, including the hippocampus and thalamus^58-60^. Separate mouse models have discovered the reduced expression of 6-GABAA and a5-GABAA receptors in epilepsy and sleep deprivation, respectively^18,57^. Such findings suggest that impairment of these GABA receptors in sleep-deprived states could lead to an increased risk of seizures in patients with epilepsy.

### CONCLUSION

This paper reviewed the possibility that reduced GABAmediated tonic inhibition could represent the mechanism by which sleep deprivation can lower the seizure threshold. Much of the literature in this field thus far supports a direct correlation between sleep deprivation and seizure risk in epilepsy. In addition, numerous studies have emphasized the diagnostic importance of clinically induced sleep deprivation. Despite these advancements, more mechanistic clinical studies remain to be done. Encouragingly, murine studies have shown lower 6-GABAA and a5-GABAA receptor expression in both epilepsy and sleep deprivation. Researchers will need to determine if these findings can be extrapolated to humans.

Additionally, these preclinical studies have investigated GABA receptor expression in sleep deprivation and epilepsy separately, but not in the same experimental setting. Future studies could, for instance, explore how reduced expression of 6-GABAA and a5-GABAA receptors in the sleep-deprived state contributes to a higher risk of seizures in people with epilepsy. Before this interplay can be addressed experimentally, however, more research needs to document and confirm the finding that 6-GABAA and a5-GABAA receptors have lower expression in sleep-deprived humans.

Sleep deprivation leading to an increased risk of epileptic seizures remains an underexplored area of research. This review addressed the paucity of mechanistic and explanatory findings by highlighting existing preclinical and clinical studies. Even if it turns out that reduced GABA-mediated inhibition is not the only factor contributing to sleep deprivation-induced seizures, elucidating this mechanism is imperative to better understand epileptogenesis, which would lead to more efficacious treatment for epilepsy.
